# Supplementary material for: Content-rich biological network constructed by mining PubMed abstracts
Source: BMC Bioinformatics. 2004 Oct 8;5:147. doi: 10.1186/1471-2105-5-147 (PMC528731; doi:10.1186/1471-2105-5-147)
Supplement: Additional File 2 — The original results of the above study (non-essential files are deleted to keep the file size under the limit set by BMC bioinformatics). [file 1471-2105-5-147-S2.bz2 › chilibotAdditionalFile2/dip10/637ID10917520E11024/html/FCER1A_IGHE.html]

 


 **FCER1A** and **IGHE** 
  
Found 14 abstracts in PubMed, retrieved 10.  
 

 What does Google say? 
 PDF only 
| .edu only 

---

**Interactive relationship** (e.g. stimulation, inhibition, etc)

**Neutral relationship**- The recent elucidation of the tridimensional structure of the complex between IgE  [ **IGHE** ]  and FcERI  [ **FCER1A** ]  provides unexpected information regarding the mechanism of assembly of the complex, which now can be used to design small chemical compounds capable of specifically inhibiting this interaction.  Ref: 12836193 Med Sci Paris , 2003
- The alpha subunit of the FcERI  [ **FCER1A** ]  binds IgE  [ **IGHE** ]  with high affinity.  Ref: 2148316 J Biol Chem, 1990
- The alpha subunit of the human IgE  [ **IGHE** ]  receptor FcERI  [ **FCER1A** ]  is sufficient for high affinity IgE  [ **IGHE** ]  binding.  Ref: 2148316 J Biol Chem, 1990
- Interaction of an allergen with its specific IgE  [ **IGHE** ]  bound to FcERI  [ **FCER1A** ]  triggers cell activation, which induces the release of numerous mediators that are responsible for allergic manifestations.  Ref: 12836193 Med Sci Paris , 2003
- This four YAC contig includes other genes with immune functions including the  **FCER1A**  gene, which encodes the alpha subunit of the IgE  [ **IGHE** ]  high affinity Fc receptor and the IFI 16 gene, an interferon gamma induced gene.  Ref: 8613143 Immunogenetics, 1996
- The recent observation that eosinophils expressed IgE  [ **IGHE** ]  binding molecules belonging to different gene superfamilies CD23, Mac2 epsilon BP and FceRI  [ **FCER1A** ] , as well as two different IgA receptors Fc alpha R, and secretory component binding site, participating both in antiparasite immune defence and in inflammatory processes, reinforces the concept of the functional duality of eosinophils, specially in tissues.  Ref: 7994628 C R Seances Soc Biol Fil, 1994
- This antibody recognises specifically free but not FcERI  [ **FCER1A** ]  bound IgE  [ **IGHE** ]  thus preventing ligand binding and subsequent cell activation.  Ref: 12836193 Med Sci Paris , 2003

**Non-interactive relationship** (e.g. studied together, co-existance, homology, etc.)

- The results of this study suggest that FcERI  [ **FCER1A** ]  IgG and MaE11 may have important immunotherapeutic benefit for the amelioration of IgE  [ **IGHE** ]  mediated diseases.  Ref: 7525678 J Allergy Clin Immunol, 1994
- Mast cells and basophils express the high affinity IgE  [ **IGHE** ]  receptor FcERI  [ **FCER1A** ]  whereas the low affinity receptor for monomeric IgE  [ **IGHE** ]  FcE RII is present on macrophages, lymphocytes, eosinophils, platelets and Langerhans cells.  Ref: 1690590 C R Acad Sci III, 1990
- This ELISA used FcERI  [ **FCER1A** ]  IgG for capture and peroxidase labelled goat polyclonal antibody to human IgE  [ **IGHE** ]  for detection.  Ref: 8760275 Mol Immunol< MedlineTA>Mol Immunol, 1990
- Human FcERI  [ **FCER1A** ]  IgG and humanized anti IgE  [ **IGHE** ]  monoclonal antibody MaE11 block passive sensitization of human and rhesus monkey lung.  Ref: 7525678 J Allergy Clin Immunol, 1994
- In this study we assessed the capacity of two inhibitors, FcERI  [ **FCER1A** ]  IgG, an immunoadhesin made up of the alpha chain of the high affinity IgE  [ **IGHE** ]  receptor joined to a truncated IgG heavy chain, and MaE11, a humanized murine anti human IgE  [ **IGHE** ]  antibody, to prevent allergen sensitization.  Ref: 7525678 J Allergy Clin Immunol, 1994
- We synthesized various analogues of indolyl, naphthyl and phenylethyl substituted halopyridyl, thiazolyl and benzothiazolyl thioureas and examined their in vitro effects on the high affinity IgE  [ **IGHE** ]  receptor FcERI  [ **FCER1A** ]  mediated mast cell leukotriene release.  Ref: 12565956 Bioorg Med Chem Lett, 2003
- Aggregation of the high affinity receptor for IgE  [ **IGHE** ]  FceRI  [ **FCER1A** ]  on mast cells results in the rapid phosphorylation of tyrosines on the beta and gamma chains of the receptor by the Src family kinase Lyn, which initiates the signaling cascades leading to secretion of inflammatory mediators.  Ref: 11388899 J Biochem Tokyo , 2001
- The high affinity receptor for IgE  [ **IGHE** ] , FcERI  [ **FCER1A** ] , is at the core of the allergic reaction.  Ref: 12836193 Med Sci Paris , 2003
- This process facilitates the cell surface expression of several proteins including the high affinity IgE  [ **IGHE** ]  receptor alpha subunit, FcERI  [ **FCER1A** ]  alpha, which otherwise requires at least one other subunit for surface expression.  Ref: 1368234 Biotechnology N Y , 1992
